# Electricity-producing *Staphylococcus epidermidis* counteracts *Cutibacterium acnes*

**DOI:** 10.1038/s41598-021-91398-7

**Published:** 2021-06-07

**Authors:** Shinta Marito, Sunita Keshari, Supitchaya Traisaeng, Do Thi Tra My, Arun Balasubramaniam, Prakoso Adi, Ming-Fa Hsieh, Deron Raymond Herr, Chun-Ming Huang

**Affiliations:** 1 Department of Biomedical Sciences and Engineering, National Central University, Taoyuan, Taiwan; 2 Department of Life Sciences, National Central University, Taoyuan, Taiwan; 3 Department of Biomedical Engineering, Chung Yuan Christian University, Taoyuan, Taiwan; 4 Department of Biology, San Diego State University, San Diego, USA

**Keywords:** Microbiology, Pathogenesis

## Abstract

*Staphylococcus epidermidis* (*S. epidermidis*) ATCC 12228 was incubated with 2% polyethylene glycol (PEG)-8 Laurate to yield electricity which was measured by a voltage difference between electrodes. Production of electron was validated by a Ferrozine assay. The anti-*Cutibacterium acnes* (*C. acnes*) activity of electrogenic *S. epidermidis* was assessed in vitro and in vivo. The voltage change (~ 4.4 mV) reached a peak 60 min after pipetting *S. epidermidis* plus 2% PEG-8 Laurate onto anodes. The electricity produced by *S. epidermidis* caused significant growth attenuation and cell lysis of *C. acnes*. Intradermal injection of *C. acnes* and *S. epidermidis* plus PEG-8 Laurate into the mouse ear considerably suppressed the growth of *C. acnes.* This suppressive effect was noticeably reversed when cyclophilin A of *S. epidermidis* was inhibited, indicating the essential role of cyclophilin A in electricity production of *S. epidermidis* against C*. acnes.* In summary, we demonstrate for the first time that skin *S. epidermidis*, in the presence of PEG-8 Laurate, can mediate cyclophilin A to elicit an electrical current that has anti-*C. acnes* effects. Electricity generated by *S. epidermidis* may confer immediate innate immunity in acne lesions to rein in the overgrowth of *C. acnes* at the onset of acne vulgaris.

## Introduction

Microbes in the human skin microbiome play a vital role in modulating both the cutaneous innate immunity and adaptive immunity, while maintaining the skin homeostasis. Dysbiosis of this system may actively influence cutaneous immunity^1,2^. Inflammatory acne vulgaris is a canonical example of a dysbiotic microbiome, where patients exhibit a striking alteration in bacterial abundance in acne lesions^3–5^. Although it has been reported that the relative abundances of *Cutibacterium acnes* (*C. acnes*), formally named as *Propionibacterium acnes*, were similar in the pilosebaceous units of healthy individuals and acne patients^6^, overgrowth of certain phylotypes of *C. acnes* correlates with the development of inflammatory acne vulgaris, which affects a 94–95% of the pubertal population, 20–40% of adults and < 25% of women worldwide^7^. Our previous studies have demonstrated that *Staphylococcus epidermidis* (*S. epidermidis*) can fermentatively metabolize molecules with multiple carbon atoms such as sucrose or polyethylene glycol (PEG)-based polyether to produce short-chain fatty acids (SCFAs)^8^. Several SCFAs including acetic, butyric, lactic, and succinic acids exert robust antimicrobial activities against the growth of *C. acnes*^9^. Carbon-rich carbohydrates and SCFAs, especially acetic acid and butyric acid, have been recognized as redox mediators for electricity production of bacteria^10–12^. Here we investigate whether *S. epidermidis* can metabolize carbon-rich molecules to yield electricity.

Electricity-producing bacteria in the environment, such as Gram-negative *Shewanella*, *Geobacter* and *Pseudomonas* species, can mediate the formation of biofilms to generate electrons and engage in the process of extracellular electron transfer (EET) to transport electrons from the bacterial cytosol to the extracellular space. This facilitates the cycling of minerals and biomaterials in nature^13,14^. Several genes encoding multiheme cytochrome C are expressed by Gram-negative bacteria to mediate electricity production^15^. Gram-positive bacteria express a single protein, called peptide pheromone encoding lipoprotein A (PplA), which contains two flavin molecules enabling electrons to exit the membrane to reach the cell’s exterior^13,14,16^. The lactic acid bacteria are Gram-positive fermenting bacteria that have electrogenic properties due to the expression quinol oxidase or quinone reductase in the EET^17^. During the process of flavin-based EET, the type II NADH dehydrogenase (Ndh2) transfers electrons from adenine dinucleotide (NAD) to demethylmenaquinone (DMK) then to flavin mononucleotide (FMN) groups on PplA or free flavin shuttle molecules^17^. Cytochrome C in Gram-negative bacteria acts as an electron donor or acceptor to shuttle electrons between its reductase and oxidase^18^. It has been documented that cytochrome C can be sequestered by cyclophilin A, a ubiquitously distributed protein with peptidyl prolyl cis–trans isomerase (PPIase) activity^19^. The binding of cyclophilin A to peroxiredoxin proteins supports its peroxidase activity as an immediate electron donor^20^. Both electrogenic *Shewanella* and *Geobacter* can produce sufficient amounts of cytochrome C to mediate their electron transfer^21^. However, electron transfer mediators in Gram-positive bacteria, which do not synthesize soluble cytochrome C, have not been characterized. Cyclophilin A is critical for bacterial growth and biofilm formation^22^ and may play a role in mediating electron transfer in Gram-positive bacteria.

Although the physiological function of electrons produced by bacteria in humans remains largely unknown, recent studies revealed that human gut bacteria including *Listeria monocytogenes*, *Enterococcus faecalis*, *Klebsiella pneumonia*, *Escherichia coli,* and many probiotic Lactobacillus species, express the genes encoding proteins responsible for growth promoting EET^23–25^. Previous studies have shown that an electric field generated by insulated electrodes can effectively inhibit the growth of *Staphylococcus aureus* and *Pseudomonas aeruginosa*^26^. This low-energy electron irradiation has been used to inactivate *Escherichia coli* and viruses due to damage to their nucleic acids^27,28^ and cell membranes^27,29^. By using *S. epidermidis* ATCC 12228, a non-biofilm forming strain, we found that *S. epidermidis* can utilize a PDG ester of lauric acid containing 50 carbon atoms to yield electricity, demonstrating non-biofilm-mediated electricity production from human bacteria^30,31^. Both *S. epidermidis* and *C. acnes* can be isolated from a single skin lesion in patients with acne vulgaris^32^. Previously, a genomic basis of antagonism between *S. epidermidis* and *C. acnes* has been proposed^32^. Here we investigate the interference of electrogenic *S. epidermidis* with *C. acnes *in vitro and in vivo. Our results demonstrate a novel mechanism by which *S. epidermidis* inhibits the growth of *C. acnes* through the production of a weak electrical current, thus identifying a previously unknown process underlying bacterial homeostasis in the skin microbiome.

## Methods

### Ethics statement

This research was carried out in strict accordance with an approved Institutional Animal Care and Use Committee (IACUC) protocol at National Central University (NCU), Taiwan (NCU-106-016) and in compliance with the Arrive guidelines (https://arriveguidelines.org/).

### Bacterial culture

*S. epidermidis* (ATCC 12,228) was cultured in tryptic soy broth (TSB) (Sigma, St. Louis, MO, USA). *C. acnes* (ATCC 6919) was cultured on Reinforced Clostridium Medium (RCM, Oxford, Hampshire, England) under anaerobic conditions using a Gas-Pak (BD Biosciences, San Jose, CA, USA). Bacteria were cultured at 37 °C until the logarithmic growth phase. Bacterial pellets were harvested by centrifugation at 5000 × g for 10 min, washed in phosphate-buffered saline (PBS), and then suspended in PBS or TSB for further experiments.

### Fermentation of bacteria

*S. epidermidis* [10^7^ colony-forming unit (CFU)/mL] was incubated in 10 ml rich media (10 g/L yeast extract (Biokar Diagnostics, Beauvais, France), 3 g/L TSB, 2.5 g/L K_2_HPO_4_, and 1.5 g/L KH_2_PO_4_) with and without 2% (20 g/L) of polyoxyethylene glycol 400 monolaurate designated as PEG-8 Laurate (C_28_H_56_O_10_) by The International Nomenclature of Cosmetic Ingredients (INCI) (Taiwan NJC Corporation, Ltd, Chiayi, Taiwan). In some experiments, *S. epidermidis* was pretreated with 1 μM TMN 355 (UNI-ONWARD, New Taipei, Taiwan)^33^, under aerobic conditions at 37 °C with shaking at 200 rpm for 12 h before adding into rich media. The 0.002% (w/v) phenol red (Sigma) in rich media acted as a fermentation indicator. A color change from red–orange to yellow indicated the occurrence of bacterial fermentation which was detected by optical density (OD)_560_ nm. To determine if PEG-8 Laurate affects the bacterial growth, bacteria (10^7^ CFU) was incubated with 2% PEG-8 Laurate in TSB media for 24 h. Bacteria were diluted 1: 10^0^ −1: 10^5^ into PBS and 10 μl from each dilution was spotted onto a TSB agar plate to count CFU. Plates were incubated at 37 °C for 12 h for *S. epidermidis* and 72 h for *C. acnes* to count the colony numbers.

### Detection of bacterial electricity

The voltage difference between the electrodes (cathode and anode) was used to detect the bacterial electricity in vitro. A carbon felt (2.5 cm × 10 cm) and a carbon cloth (10 cm × 10 cm) (Homy Tech, Taoyuan, Taiwan) were used as an anode and a cathode, respectively. The carbon cloth was wrapped up in a nafion membrane N117 (6 cm × 6 cm) (Homy Tech), a proton exchange membrane (PEM), and placed in a 10 cm diameter petri dish. Anode and cathode were connected by copper wires, which in turn were bridged to external resistance of 200 Ω. Bacteria (S*. epidermidis* or *C. acnes*) cultured overnight to 10^7^ CFU in rich media with/without 2% PEG-8 Laurate (200 μL) were pipetted onto the surface of the anode. In some experiments, *S. epidermidis* was pretreated with 1 μM TMN 355 for 12 h before pipetting onto the anode. The voltage difference (mV) against time (min) was monitored by a digital multimeter (Lutron, DM-9962SD, Sydney, Australia). The voltage was recorded every 10 s to plot a graph of voltage against time^34^.

### The interference of *S. epidermidis* with the growth of *C. acnes *in vitro

*C. acnes* ATCC 6919 (10^7^ CFU) was incubated with 0.22 µm-filtered supernatants obtained from the culture of *S. epidermidis* (10^7^ CFU) in the presence or absence of 2% PEG-8 Laurate for 60 or 300 min. After incubation for 1 h, *C. acnes* was serially diluted and spotted onto a *C. acnes* selective agar plate to count CFU. In other experiment, rich media containing *C. acnes* ATCC 6919 (10^7^ CFU) was added into a 10 cm diameter petri dish where a carbon felt, a carbon cloth and PEM were placed. *S. epidermidis* (10^7^ CFU) pretreated with/without 1 µM TMN 355 in rich media supplemented with/without 2% PEG-8 Laurate was pipetted on the surface of the anode. After that, *C. acnes* was collected from the petri dish, serially diluted, and spotted onto a selective agar plate containing rich media plus furazolidone (10 µg/mL) to count CFU. Results in our previous publication^9^ have demonstrated that furazolidone (10 µg/mL) in a selective agar plate completely inhibits the growth of *S. epidermidis*, but not *C. acnes*. To measure the membrane permeability^35^, *C. acnes* was suspended in 0.5 mM PBS (pH 7.4) containing 10 µg/mL crystal violet (Sigma) followed by centrifugation at 9300 × g for 10 min. After further incubation at 37 °C for 10 min, the suspension was centrifuged at 13,400 × g for 15 min. The OD_590_ of the supernatant was measured using untreated *C. acnes* as a blank. The OD_590_ value of 10 µg/mL crystal violet solution was considered as 100%. The percentage of crystal violet uptake was calculated as follows: (OD_590_ value of sample/ OD_590_ value of crystal violet solution) × 100 = Percentage uptake of crystal violet.

### High-performance liquid chromatography (HPLC) analysis

Cultured media of *S. epidermidis* (10^7^ CFU) with/without 2% PEG-8 Laurate after 60 or 300 min of incubation were centrifuged at 5000 rpm for 10 min. The supernatants were filtered through a 0.22 μm microfiltration membrane and butyric acid in supernatants was detected according to the previous protocol^36^. The concentrations of butyric acid were quantified based on a calibration curve of a butyric acid analytical standard.

### Ferrozine assays

Pellets from *S. epidermidis* (10^7^ CFU) pretreated with/without 1 μM TMN 355 were suspended in TSB media supplemented with/without 2% PEG-8 Laurate and 4 mM ferrozine (Alfa Aesar Chemicals, Tewsbury, MA, USA). An equal volume of 50 mM ferric ammonium citrate (Sigma) with 100 μL of bacteria in a 96-well plate was incubated for 1 h at 37 °C. Media with *S. epidermidis* alone or PEG-8 Laurate alone in the presence of ferrozine and ferric ammonium citrate were used as negative controls. The color change of media containing ferrozine and ferric ammonium citrate was measured at OD_562_ and quantified by a calibration curve^16^.

### *S. epidermidis* against *C. acnes* in vivo

Institute of Cancer Research (ICR) female mice (8–9 weeks old) purchased from the National Laboratory Animal Center, Taipei, Taiwan were anesthetized by isoflurane (Sigma). Five mice per group were used in each experiment. The ears of ICR mice were injected intradermally with *C. acnes* (ATCC 6919) (10^7^ CFU) and *S. epidermidis* (ATCC 12228) (10^7^ CFU) with/without 2% PEG-8 Laurate using an insulin syringe with 29 G × 1/2 inches (BD Biosciences, San Jose, CA, USA). The mouse ears co-injected with *C. acnes* and *S. epidermidis* pretreated in the presence and absence of 1 μM TMN 355 for 12 h were included as controls. After 24 h, ears were excised and homogenized for bacterial counts. Ear homogenates were made by a tissue grinder in 200 μL of sterile PBS. CFUs of *C. acnes* in ear homogenates were enumerated by plating serial dilutions (1:10^0^–1:10^5^) of homogenates on *C. acnes* selective agar plates containing rich media and 10 μg/mL of furazolidone (Sigma)^37^. Plates were incubated for 72 h at 37 °C under anaerobic conditions using a Gas-Pak.

### Statistical analysis

Experiments were repeated at least three times to ensure reproducibility. Data are presented as mean values ± standard deviation (SD). Statistical significance was determined using Student’s unpaired two-tailed t-test, as indicated in the legend (*P < 0.05, **P < 0.01, ***P < 0.001 and ns = non-significant).

## Results

### PEG-8 Laurate induces electricity production by *S. epidermidis*

**Figure 1:**
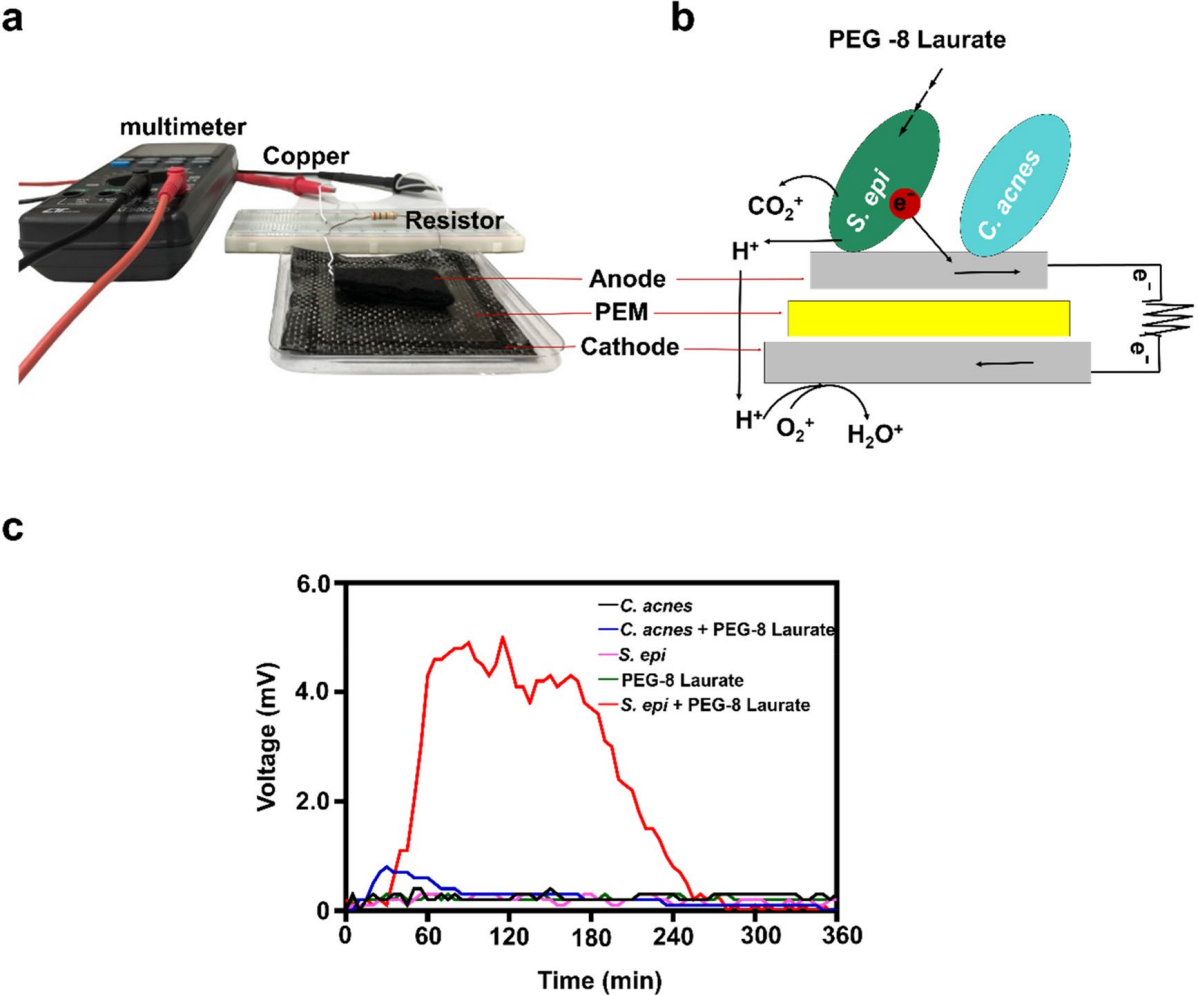
PEG-8 Laurate triggered *S. epidermidis* to produce electricity measured by voltage changes. (**a**) The surface of anode (a carbon felt) was spotted with *S. epidermidis* ATCC 12228 (*S. epi*) (10^7^ CFU) or *C. acnes* ATCC 6919 (10^7^ CFU). PEM (a nafion membrane) was used to separate the anode and cathode (a carbon cloth) which were connected via copper wires to a conductive resister. The mV was detected by a digital multimeter. (**b**) Schematic of diagram represented in vitro detection of bacterial electricity. Electrons produced by *S. epidermidis* in the presence of PEG-8 Laurate on anode were transferred to cathode. (**c**) The voltage difference (mV) between anode and cathode in the presence of *S. epidermidis* or *C. acnes* with or without PEG-8 Laurate was monitored for 360 min. Illustration (**a**, **b**) is from own resources.

Bacteria develop biofilms on microbial fuel cell (MFC) electrodes, enhancing electricity production during the EET process^38^. The biofilm matrix produced by the bacteria is conductive, which allows electrons to move efficiently to the electrodes. Addition of organic acids such as acetic acid generates electrons by oxidation of organic acids by bacteria. Here we investigate the electrogenicity of *S. epidermidis* ATCC 12228, a non-biofilm forming skin bacterium, in the presence of 2% PEG-8 Laurate, a carbon-rich PEG ester of lauric acid. *S. epidermidis* 10^7^ CFU and 2% PEG-8 Laurate in rich media were added onto the surface of the anode. Addition of the same volume of *S. epidermidis* (10^7^ CFU) alone or 2% PEG-8 Laurate alone acted as controls. As shown in Fig. 1, little or no voltage change was detected in media containing *S. epidermidis* or PEG-8 Laurate alone throughout the monitoring period of 360 min. However, a marked increase in voltage to 4.4 mV was detected when 2% PEG-8 Laurate was added to media containing *S. epidermidis.* Voltage production peaked at 60 min, and remained steady for 180 min before declining to a baseline voltage of 0.2 mV at 300 min. This result clearly illustrates the biofilm-independent electrogenic properties of skin *S. epidermidis* in the presence of PEG-8 Laurate.

### Electrogenic *S. epidermidis* impedes the growth of *C. acnes*

**Figure 2:**
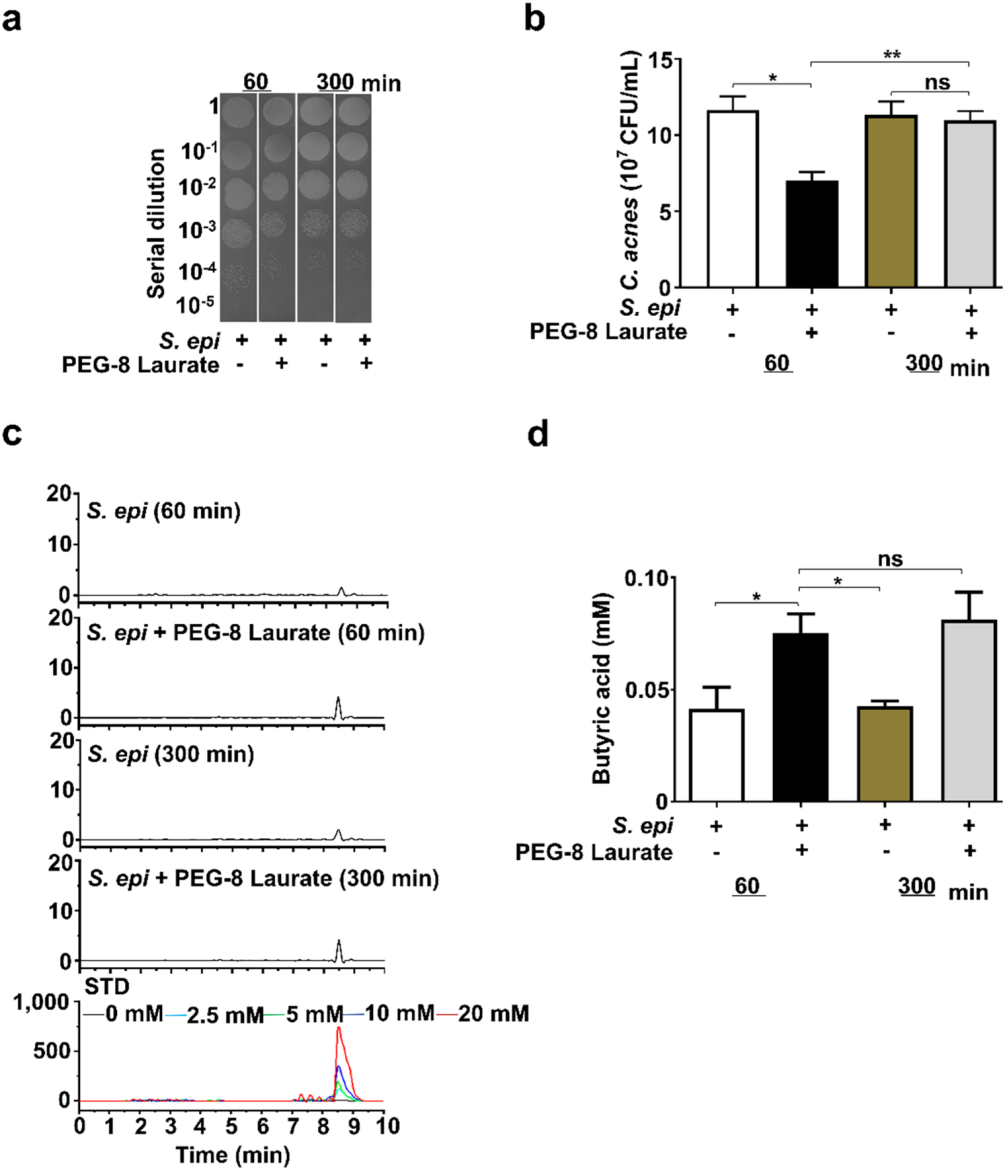
High production of electricity, not butyric acid, contributed to anti-*C. acnes* effect of *S. epidermidis* plus PEG-8 Laurate. (**a**) *C. acnes* ATCC 6919 (10^7^ CFU) was incubated with media collected from the culture of *S. epidermidis* (*S. epi*) in the presence or absence of PEG-8 Laurate for 60 min or 300 min. *C. acnes* was serially diluted (1: 10^0^ −1: 10^5^) and spotted on a TSB plate for CFU counts. (**b**) The graph represents the CFU/mL of *C. acnes* from three independent experiments. (**c**) Representative chromatography of HPLC analysis of butyric acid in media collected from the culture of *S. epidermidis* in the presence or absence of PEG-8 Laurate for 60 min or 300 min. (**d**) The concentration (mM) of butyric acid was quantified based on the heights [milli-absorbance unit (mAU)] of standard (STD) peaks with concentrations of butyric acid from 0–20 mM. Data are represented as mean ± SD). *P < 0.05 (two-tailed t-tests). ns = non-significant.

Since both *S. epidermidis* and *C. acnes* are bacterial members in an acne microbiome^39^, we sought to assess if electricity generated by *S. epidermidis* in the presence of PEG-8 Laurate can alter the growth of *C. acnes*. The culture media of *S. epidermidis* plus PEG-8 Laurate which elicited the high and low voltage peaks at 60 and 300 min, respectively (Fig. 1), were added into a culture of *C. acnes* for 60 min. The incubation of *C. acnes* with media of *S. epidermidis* alone was included as a control. As shown in Fig. 2a,b, the number (7.0 ± 0.5 × 10^7^ CFU) of *C. acnes* incubated with media collected from 60 min after the culture of *S. epidermidis* plus PEG-8 Laurate was significantly lower than that (11.6 ± 0.8 × 10^7^ CFU) incubated with media collected from in the culture of *S. epidermidis* alone. Higher *C. acnes* counts were detected when *C. acnes* incubated with media collected from 300 min after culture of *S. epidermidis* in the presence (11.0 ± 0.5 × 10^7^ CFU) or absence (11.3 ± 0.8 × 10^7^ CFU) of PEG-8 Laurate. This result suggests that high electricity in media collected from 60 min after culture of *S. epidermidis* plus PEG-8 Laurate hindered the growth of *C. acnes*.

Our previous studies have demonstrated that *S. epidermidis* can mediate fermentation to produce SCFAs including butyric acid to suppress the growth of *C. acnes*^40^. We next measured the production of butyric acid in the culture media of *S. epidermidis* with/without PEG-8 Laurate for 60 and 300 min by HPLC. As shown in Fig. 2c,d, the levels of butyric acid in media of *S. epidermidis* plus PEG-8 Laurate for both 60 and 300 min cultures are higher than those in media of *S. epidermidis* alone. However, there is no difference in the level (approximately 0.08 mM) of butyric acid in media of the culture of *S. epidermidis* plus PEG-8 Laurate for either 60 or 300 min. Furthermore, PEG-8 Laurate itself did not affect the growth of both *S. epidermidis* and *C. acnes* (Fig. S1). These results suggested that high electricity produced from 60 min-culture of *S. epidermidis* plus PEG-8 Laurate, neither butyric acid nor PEG-8 Laurate itself, contributed to the suppressive effect of *S. epidermidis* against *C. acnes*.

### The blockade of electricity production attenuates the suppressive effect of *S. epidermidis* against *C. acnes *in vitro

**Figure 3:**
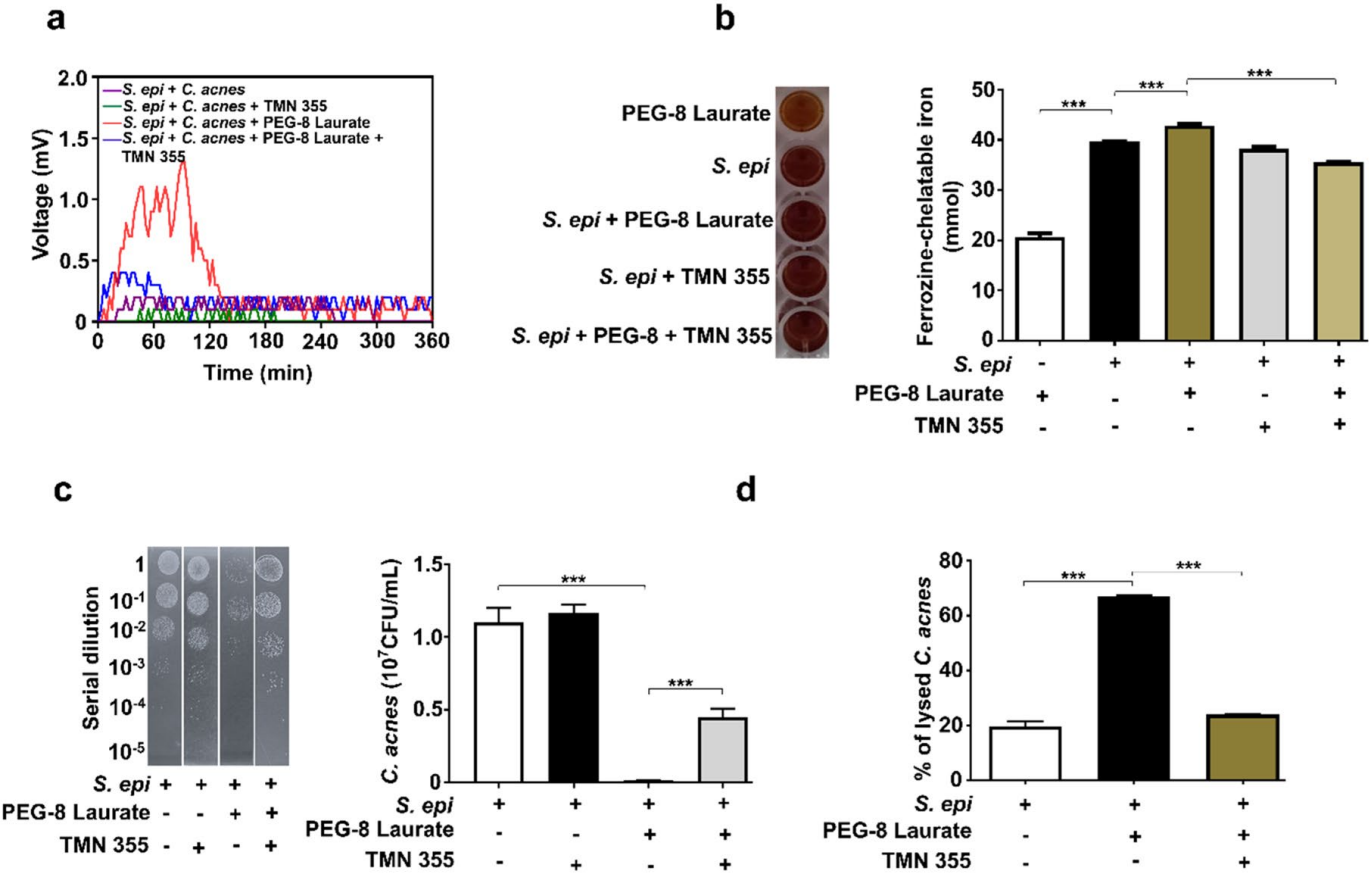
Electrons produced by *S. epidermidis* plus PEG-8 Laurate caused lysis of *C. acnes*. (**a**) The voltage differences (mV) which were measured in conditions of anodes pipetted with media containing *S. epidermidis* (*S. epi*) pretreated with/without TMN 355 in the presence or absence of PEG-8 Laurate, and *C. acnes* were placed in a petri dish containing anode, cathode and PEM. (**b**) Electrons produced by *S. epidermidis* plus PEG-8 Laurate were detected using a ferrozine assay. *S. epidermidis* pretreated with/without TMN 355 was added into TSB media supplemented with ferrozine and ferric ammonium citrate in the presence or absence of PEG-8 Laurate. Addition of PEG-8 Laurate alone into TSB media supplemented with ferrozine and ferric ammonium citrate served as a control. The formation (dark brown) of ferrozine-chelatable irons (mM) was quantified by OD_562_ measurements. (**c**) *C. acnes* in anodes was collected and serially diluted (1 : 10^0^–1 : 10^5^) before spotting on a selective agar plate consisting of rich media plus furazolidone (10 µg/mL). The graph represents the CFU/mL of *C. acnes* from three separate experiments. (**d**) The percentage (%) of lysed *C. acnes* assayed by the uptake of crystal violet was displayed after response of *C. acnes* in cathodes to anodes pipetted with *S. epidermidis* or TMN 355-pretreated *S. epidermidis* in the presence or absence of PEG-8 Laurate. Data are represented as mean ± SD (n = 3 each group). ***P < 0.001, (two-tailed t-tests).

To examine the essential role of electricity produced by *S. epidermidis* in the growth inhibition of *C. acnes*, *S. epidermidis* was pretreated with 1 µM TMN 355, a potent inhibitor of cyclophilin A, for 12 h. To confirm that this treatment prevents electricity generation, *S. epidermidis* pretreated with/without TMN 355 in the presence or absence of PEG-8 Laurate in rich media was pipetted on the surface of an anode. Electron produced by *S. epidermidis* plus PEG-8 Laurate can instantly interact with *C. acnes* which was placed in rich media in a 10 cm diameter petri dish containing anode, cathode and PEM. As shown in Fig. 3a, a high peak of voltage at approximately 1.3 mV was detected when *S. epidermidis* plus PEG-8 Laurate, not *S. epidermidis* alone, was pipetted on anode. Blocking cyclophilin A of *S. epidermidis* significantly reduced the voltage (~ 0.1 mV) induced by PEG-8 Laurate. Furthermore, inhibition of *S. epidermidis* cyclophilin A by TMN 355 did not influence the activity of PEG-8 Laurate fermentation of *S. epidermidis* (Fig. S2). This result demonstrates that cyclophilin A is an essential mediator of electricity production in *S. epidermidis*.

### Production of electrons by *S. epidermidis* is detected by ferrozine assays and induces the lysis of *C. acnes*

In the presence of electrons, ferric (Fe^3+^) ammonium citrate is converted to ferrozine-cheltable irons (dark brown) which can be quantified by measurement of OD_562_. As shown in Fig. 3b, S*. epidermidis* plus PEG-8 Laurate induced a higher amount of ferrozine-cheltable irons than *S. epidermidis* alone. Inhibition of cyclophilin A by TMN 355 considerably lowered the amount of ferrozine-cheltable irons induced by *S. epidermidis* plus PEG-8 Laurate. These data confirmed the production of electrons by *S. epidermidis* in the presence of PEG-8 Laurate. After pipetting *S. epidermidis* with/without PEG-8 Laurate on an anode for 360 min, *C. acnes* in a petri dish containing an anode and a cathode were collected and spotted on a furazolidone-supplemented selective agar plate to determine if electrons influence the growth of *C. acnes*. Results in Fig. 3c demonstrate that a two log_10_ reduction in the number (1.1 ± 0.1 × 10^7^ CFU) of *C. acnes* was detected when an anode was coated with *S. epidermidis* plus PEG-8 Laurate compared to that (1.2 ± 0.04 × 10^5^ CFU) obtained from an anode coated with *S. epidermidis* alone. To examine if membranes of *C. acnes* are compromised by electrons generated by *S. epidermidis* plus PEG-8 Laurate, *C. acnes* were collected following the treatment in Fig. 3c and were stained with crystal violet to visualized lysed cells. As shown in Fig. 3d, treatment with *S. epidermidis* plus PEG-8 Laurate, not *S. epidermidis* alone, resulted in a noticeable increase in the percentage of lysed *C. acnes.* These results demonstrate that electrons produced by *S. epidermidis* plus PEG-8 Laurate can suppress the growth of C. *acnes* by disruption of membrane integrity. Inhibition of *S. epidermidis* cyclophilin A by TMN 355 significantly weakened this effect (Fig. 3c,d), illustrating a cyclophilin A-mediated pathway of electricity production in *S. epidermidis* against *C. acnes*.

### Electron production mediated by cyclophilin A in *S. epidermidis* attenuates viability of *C. acnes *in vivo

**Figure 4:**
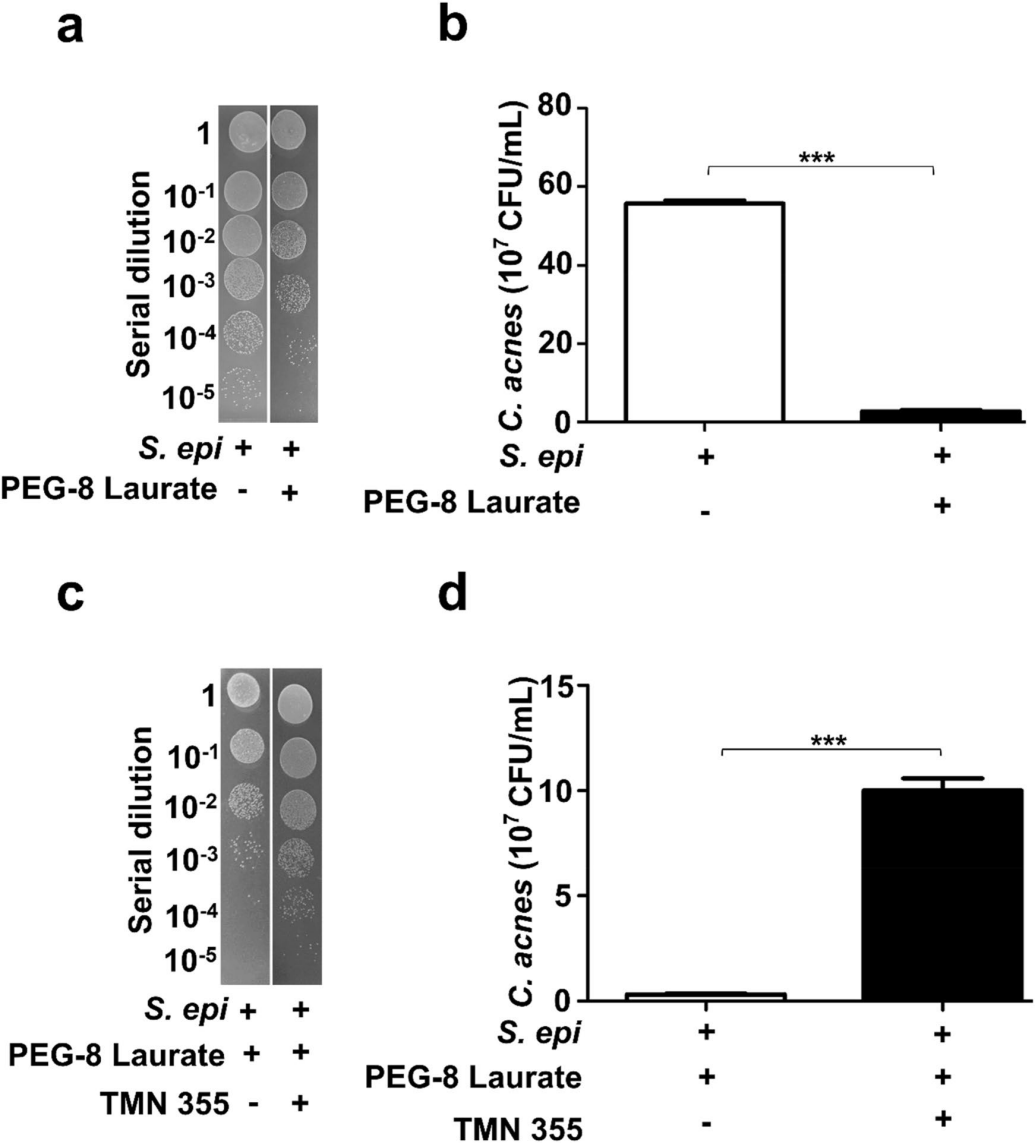
Cyclophilin A is an essential component for the interference of electrogenic *S. epidermidis* with *C. acnes *in vivo. (**a**) The number of *C. acnes* in ears of ICR mice after co-injection of *C. acnes* (10^7^ CFU) and *S. epidermidis* (*S. epi*) (10^7^ CFU) in the presence or absence of 2% PEG-8 Laurate was quantified after 72 h. (**b**) The number of *C. acnes* in ears of ICR mice after co-injection of *C. acnes* and *S. epidermidis* pretreated with/without TMN 355 in the presence of 2% PEG-8 Laurate was calculated after 72 h. The CFUs of *C. acnes* in mouse ears were enumerated by plating serial dilutions (1 : 10^0^ −1 : 10^5^) of the ear homogenate on furazolidone*-*supplemented agar plates. Data are represented as mean ± SD, in triplicate from five mice per group. ***P < 0.001 (two-tailed t-tests).

Integrating all in vitro data above supports the model that electrogenic *S. epidermidis* suppresses the growth of *C. acnes*, thus we employed a mouse ear model to investigate the counteraction of *S. epidermidis* to *C. acnes *in vivo. Ears of ICR mice were intradermally injected with *S. epidermidis* and *C. acnes* in the presence or absence PEG-8 Laurate for 24 h. As shown in Fig. 4a,b, the number (5.7 ± 0.3 × 10^7^ CFU) of *C. acnes* from mouse ears injected with *S. epidermidis* and *C. acnes* in the presence of PEG-8 Laurate was significantly lower than that (55.7 ± 0.9 × 10^7^ CFU) injected with *S. epidermidis* and *C. acnes* in the absence of PEG-8 Laurate. This result suggests that PEG-8 Laurate provokes electricity production in *S. epidermidis* against *C. acnes*. An extremely low voltage difference (< 0.5 mV) was detected when *C. acnes* and PEG-8 Laurate were pipetted on an anode (Fig. 1 and Fig. S3). To verify the cyclophilin A-mediated pathway of electricity production in *S. epidermidis* against *C. acnes *in vivo, *S. epidermidis* was pretreated with or without TMN 355 before intradermally injecting into mouse ear with *C. acnes* and PEG-8 Laurate. The effect of lowering the number of *C. acnes* by *S. epidermidis* plus PEG-8 Laurate was significantly reversed when TMN 355-pretreated *S. epidermidis* was injected into mouse ear (Fig. 4c,d). These data suggest that PEG-8 Laurate-induced electricity mediated by cyclophilin A in *S. epidermidis* contributes to bacterial interference in vivo.

## Discussion

Antibiotics without bacterial selectivity for the treatment of acne vulgaris carry a risk of developing antibiotic resistant *C. acnes* and may result in dysbiosis in the skin human microbiome. Transferring electron from the cytosol to the exterior of the cell via the EET process represents an alternative strategy that may selectively target pathogenic bacteria. Media containing a high content of electricity (> 4 mV) collected from the culture of *S. epidermidis* plus PEG-8 Laurate for 60 min exerted a marked anti-*C. acnes* activity (Fig. 2a,b). Although a lower electricity (~ 1.2 mV) was detected when both *C. acnes* and *S. epidermidis* plus PEG-8 Laurate were simultaneously present in media (Fig. 3a,c), the anti-*C. acnes* activity of electrogenic *S. epidermidis* still remained. Although low electricity of *C. acnes* in the presence of PEG-8 Laurate was detectable (Fig. 1), electron acceptors on the cell wall of *C. acnes* may interrupt the intensity of electricity produced by *S. epidermidis*. It has been reported that electrogenesis can be influenced by different bacteria when they are co-cultured ^41^. Addition of *C. acnes* in the presence of PEG-8 Laurate onto the surface of anode created an extremely low voltage difference (< 0.5 mV) which did not affect the growth of *S. epidermidis* (Fig. S3), supporting that *S. epidermidis*, not *C. acnes*, exerted the electrogenicity and antibacterial activity when both bacteria were co-cultured in the presence of PEG-8 Laurate.

It has been reported that membrane-bound electron transport protein entrapped inside an electrochemically active biofilm facilitates electron transfer^42^. For example, electron transfer in *Shewanella oneidensis* (*S. oneidensis*) or many lactic acid bacteria in the human gut occurs directly by the formation of biofilm, as shown to occur on the surface of electrodes through extensions in the form of nano-wires^43,44^. Interestingly, an indirect electron transfer was also detected in *S. oneidensis* that involves excreting redox-active mediators such as flavin molecules that act as small diffusible shuttle molecules to transfer electron between electrodes^44^. However, the formation of biofilm is associated with the production of exopolysaccharides (EPS), quorum sensing signaling molecules and other stress factors such as heavy metal stress^45^, salinity^46^, pH^47^, nutrient starvation^48^, and pathogen invasion. Moreover, the products from microbial fermentation, such as hydrogen, formate, or acetate as redox mediators, prove an advantage over the electron transfer by the biofilm formation process^49,50^. A decrease in electron transport observed in *S. oneidensis* by inhibiting fermentation supports the model that fermentation is likely to be associated with enhancing electron transport^51^. As shown in Fig. S2, *S. epidermidis* can utilize PEG-8 Laurate to undergo fermentation which may generate SCFAs as redox mediators for electricity production. We, here, identify *S. epidermidis* ATCC 12,228 as a non-biofilm producing skin commensal bacteria which may generate electricity through PEG-8 Laurate as an electron donor^31,52^. The electricity produced by *S. epidermidis* plus PEG-8 Laurate can be enhanced by addition of FMN (Fig. S4), supporting flavin-based EET in Gram-positive bacteria^53^.

The addition of *S. epidermidis* plus PEG-8 Laurate induced a higher voltage (4.4 mV) within 60 min, which remained elevated for ~ 120 min, before declining to baseline by 300 min (Fig. 1). This indicates high electron availability at 60 min, with little remaining 300 min after addition of PEG-8 Laurate into bacterial media. Incubation of *C. acnes* with media collected at 60 min induced a significantly greater reduction in *C. acnes* viability relative to the incubation of *C. acnes* with media collected at 300 min (Fig. 2). There was no statistically significant difference in the content of butyric acid present in media of *S. epidermidis* plus PEG-8 Laurate at 60 min versus 300 min after culture (Fig. 2c,d). A butyric acid concentration less than 0.1 mM was produced within a 300 min culture of *S. epidermidis* plus PEG-8 Laurate (Fig. 2c,d). Data from our previous study demonstrated that the minimum bactericidal concentration (MBC) of butyric acid for *C. acnes* was 10 Mm^54^, indicating that the amount of butyric acid produced within a 300 min culture of *S. epidermidis* plus PEG-8 Laurate was not sufficient to kill *C. acnes*. Results in Fig. 2 indicated that electrons, not butyric acid, in culture media exerted the anti-*C. acnes* property in vitro. Furthermore, direct delivery of a voltage at 4.4 mV to *C. acnes *in vitro in the absence of *S. epidermidis* plus PEG-8 Laurate reduced the growth of *C. acnes* (Fig. S5). Although the reduction (< one log_10_) of *C. acnes* growth by 4.4 mV voltage was less than that (> two log_10_) by *S. epidermidis* plus PEG-8 Laureate (Fig. 3c), the results in Fig. S5 clearly demonstrated the anti-*a. acnes* property of electricity. However, we cannot rule out the possibility that SCFAs and other fermentation metabolites potentiated the effect of the electrons to fully eradicate *C. acnes *in vivo.

Genome-based antagonism between S*. epidermidis* and *C. acnes* highlighted the expression of antimicrobial substances in *S. epidermidis* against *C. acnes*^55^. Succinate in the metabolites of glycerol fermentation of *S. epidermidis* effectively inhibited the growth of *C. acnes *in vivo^56^. The *C. acnes* phylotype IA1, a high predominance of phylotype in acne lesions, can trigger inflammatory responses including activation of Toll-like receptors (TLRs), secretion of pro-inflammatory cytokines and infiltration of immune cells^57,58^. Although we do not know whether electricity generated by *S. epidermidis* can directly activate immune cells to eliminate *C. acnes *in vivo, it has been reported that electric fields increased the phagocytosis in macrophages^59^. Inhibition of cyclophilin A in *S. epidermidis* by 1 µM TMN 355 for 12 h completely abolished the electricity production (Fig. 3a). Intradermal co-injection of *C. acnes* with TMN 355 pretreated *S. epidermidis* in the presence of PEG-8 Laurate only partially reduced the suppressive effect of *S. epidermidis* plus PEG-8 Laurate on the growth of *C. acnes *in vivo (Fig. 4c,d). Several microbes have been identified in subepidermal compartments of normal skin^60^. It is worth investigating how *S. epidermidis* pretreated with or without TMN 355 works together with other skin microbes to fully eliminate *C. acnes *in vivo. The PEG-8 Laurate fermentation activity of *S. epidermidis* still remained after pretreatment of *S. epidermidis* with TMN 355 (Fig. S2), indicating that SCFAs can be produced by TMN 355-preteated *S. epidermidis*. Both electrons produced by *S. epidermidis* and SCFA-activated skin immunity^54^ may be required for complete eradication of *C. acnes *in vivo. Electron production by PEG-8 Laurate fermentation of *S. epidermidis* may provide an immediate innate immunity to lyse *C. acnes* at an early stage of *C. acnes* overgrowth. We further show that cyclophilin A is a key mediator of electron production of *S. epidermidis*. Cyclophilin is highly conserved across genera in both prokaryotes and eukaryotes. TMN 355, a potent cyclophilin A inhibitor, can down-regulate the gene expression of cyclophilin A and Ndh2 in *S. epidermidis* (data not shown). This is not due to toxicity since the inhibition of cyclophilin A by TMN 355 does not influence bacterial fermentation or survival (Figs S1 and S2). This is in line with a previous study that identified a flavin-based EET mechanism in Gram-positive bacteria that utilizes membrane-anchored Ndh2 for electricity production^61^. Our results demonstrate for the first time that cytoplasmic cyclophilin A in *S. epidermidis* is an essential component for electricity production.

Low-intensity direct currents or low-frequency alternating electric fields use conductive electrodes to produce free radicals, modify pH or alter exopolysaccharide matrix in bacterial biofilm. The interaction of the electromagnetic field with charged particles present in that matrix causes electron-mediated bacterial cell lysis^26^. In eukaryotic cells, including cancer cells, cell death through an electric field is associated with plasma membrane permeabilization, cytochrome C release into the cytoplasm, disorientation of the spindle microtubules or internucleosomal DNA fragmentation, resulting in necrotic cell transformation^62,63^. The increase in lysis of *C. acnes* (Fig. 3d) is likely to be due to electrolysis activity generated by *S. epidermidis* in the presence of PEG-8 Laurate. This has been reported to result from several processes including cell wall degradation^64^, or inactivation or breakdown of membrane proteins involved in cellular respiration glycolysis or cell division such as glycerol-3-phosphate dehydrogenase (GPDH)^65^ or filamenting temperature-sensitive mutant Z (FtsZ)^66,67^. These proteins share the similar dipole moments with tubulin in eukaryotes^68^, which is known to undergo disruption by low electric field. In addition, exposure of bacteria to a high pulsed electric field induces plasma membrane permeabilization and disrupts cell wall integrity, leading to loss of viability^64^. In the case of Gram-positive bacteria, a high electric pulse can lead to a change in the zeta potential of the surface charge especially at the phosphoryl groups of teichoic acids, causing cell wall degradation^69^.

In summary, deciphering the electrogenic characteristics of each bacterium leads to a better understanding of complex interplay involved in the human microbiome. Here, we present a novel modality to inhibit the growth of *C. acnes* using cyclophilin A-mediated electricity generated by skin *S. epidermidis* in the presence of PEG-8 Laurate and illustrate an electric pathway for drug targeting in acne vulgaris.

## Supplementary Information

Supplementary Information.
