# Supplementary material for: Electricity-producing Staphylococcus epidermidis counteracts Cutibacterium acnes
Source: Sci Rep. 2021 Jun 7;11:12001. doi: 10.1038/s41598-021-91398-7 (PMC8184966; doi:10.1038/s41598-021-91398-7)

**Supplementary Materials**

**Electricity-producing *Staphylococcus epidermidis* Counteracts *Cutibacterium acnes***

**Shinta Marito^1^, Sunita Keshari^2^, Supitchaya Traisaeng^2^, Do Thi Tra My^1^, Arun Balasubramaniam^1^, Prakoso Adi^1^, Ming-Fa Hsieh^3^, Deron Raymond Herr^4^, and Chun-Ming Huang^1^***

**^1^Department of Biomedical Sciences and Engineering, National Central University, Taoyuan, Taiwan**

**^2^Department of Life Sciences, National Central University, Taoyuan, Taiwan**

**^3^Department of Biomedical Engineering, Chung Yuan Christian University, Taoyuan, Taiwan**

**^4^Department of Biology, San Diego State University, San Diego, USA**

***Correspondence: Professor Chun-Ming Huang, Department of Biomedical Sciences and Engineering, National Central University, Taoyuan, Taiwan. E-mail:chunming@ncu.edu.tw; Tel.: +886-3-422-7151 ext. 36101; Fax: +886-3-425-3427**

**Supplementary methods**

**The influence of voltage difference created by *C. acnes* on the growth of *S. epidermidis*.** The rich media containing *S. epidermidis* ATCC 12228 (10^7^ CFU) was added into a 10 cm diameter petri dish where anode, cathode and PEM (Fig. 1a and b) were placed. *C. acnes* (10^7^ CFU) in the presence of 2% PEG-8 Laurate or the same volume of water was pipetted on the surface of the anode. The voltage differences (mV) were measured by a digital multimeter for 360 min. After that, *S. epidermidis* was collected from the petri dish, serially diluted (1 : 10^0^ - 1 : 10^5^), and spotted onto a TSB agar plate to count CFU.

**The effect of voltage generation on the growth of *C. acnes.*** The voltage was generated by a power supply using ELITE (BioPro Scientific Co., LTd. Hsinchu, Taiwan) in constant current mode. The maximum and minimum voltages were set at 4.4 mV and -4.4 mV, respectively. The anode was connected to a lead current input (I_in_) and the cathode was linked to voltage input (V_in_) and output (V_out_). *C. acnes* (10^7^CFU/mL) was pipetted onto to the anode for 1 h. Bacteria on the anode without providing voltage were used as a control. *C. acnes* was then collected from the anode, serially diluted, and spotted onto a TSB agar plate to count CFUs.

**Figure Legends**

**Supplementary Fig. S1.** There was no effect of PEG-10 Laurate on growth of *S. epidermidis* or *C. acnes*. (**a**) *S. epidermidis* ATCC 12228 (*S. epi*) or *C. acnes* ATCC 6919 (10^7^ CFU) was incubated with and without 2% PEG-10 Laurate for 24 h. CFUs of bacteria were enumerated by plating serial dilution (1:10^0^-1:10^5^) on TSB agar plates. (**b**) Data of CFU/mL are represented as mean ± SD, in triplicate, two-tailed t-tests. ns = non-significant.

**Supplementary Fig. S2.** Inhibition of cyclophilin A did not affect the PEG-8 Laurate fermentation of *S. epidermidis.* Rich media containing phenol red were used for culture of PEG-8 Laurate alone, *S. epidermidis* (*S. epi*) alone, *S. epidermidis* plus PEG-8 Laurate, TMN 355 alone, or TMN 355-pretreated *S. epidermidis* in the presence or absence of PEG-8 Laurate for 12 h. Fermentation was detected in the culture of *S. epidermidis* pretreated with/without TMN 355 in the presence of PEG-8 Laurate. The condition that phenol red in media of turned yellow (**a**) leading to significant reduction of OD_560_ (**b**) indicated the occurrence of bacterial fermentation. Data are represented as mean ± SD from three independent experiments. *** *P* < 0.001, (two-tailed t-tests). ns = non-significant. Illustration (**a**) is from own resources.

**Supplementary Fig. S3.** Extremely low voltage difference detected by *C. acnes* plus PEG-8 Laurate did not influence the growth of *S. epidermidis*. (**a**) The voltage differences (mV) were measured for 360 min after pipetting *C. acnes* (10^7^ CFU) with PEG-8 Laurate or water (H_2_O) on the surface of anode in the presence of *S. epidermidis* (10^7^ CFU) in a petri dish containing anode, cathode and PEM. (**b**) *S. epidermidis* was collected, serially diluted (1 : 10^0^ - 1 : 10^5^), and spotted on TSB agar plates for CFU counts. Data of CFU/mL are denoted as mean ± SD, in triplicate, two-tailed t-tests. ns = non-significant.

**Supplementary Fig. S4.** Addition of FMN enhanced electricity production of *S. epidermidis*. 10 µM FMN or the same volume of sterile water (H_2_O) was added into rich media containing *S. epidermidis* (*S. epi*) ATCC 12228 (10^7^ CFU) plus 2% PEG-8 Laurate. The voltage difference (mV) was measured for 120 min after pipetting *S. epidermidis* plus PEG-8 Laurate with/without FMN on anodes.

**Supplementary Fig. S5.** Voltage difference exerts an inhibitory effect on the growth of *C. acnes*. (**a**) Voltage difference was created by a power supply in current constant model. (**b**) *C. acnes* (10^7^ CFU) on the surface of anode was provided without (0) or with 4.4 mV for 1 h. CFUs of *C. acnes* bacteria were enumerated by plating serial dilution (1:10^0^-1:10^5^) on TSB agar plates. Data of CFU/mL are displayed as mean ± SD, in triplicate, ***P* < 0.01 (two-tailed t-tests). Illustration (**a**) is from own resources.

**
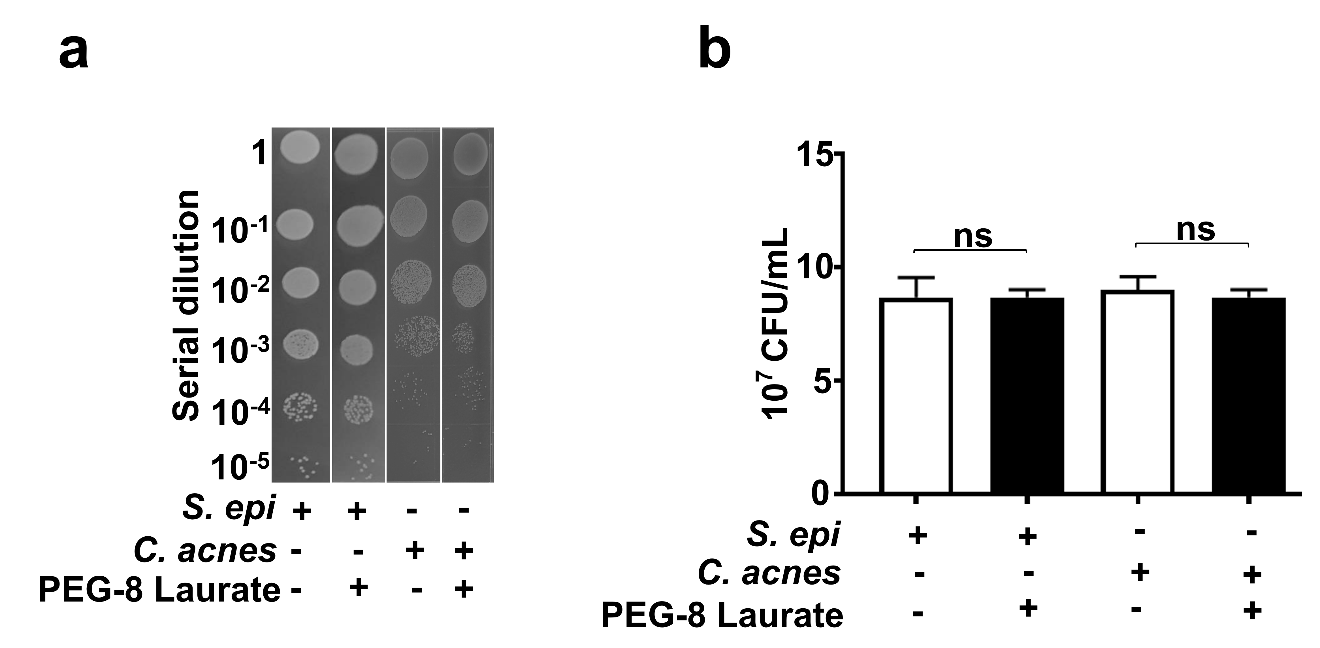
Supplementary Fig. S1.**

**Supplementary Fig. S2.**


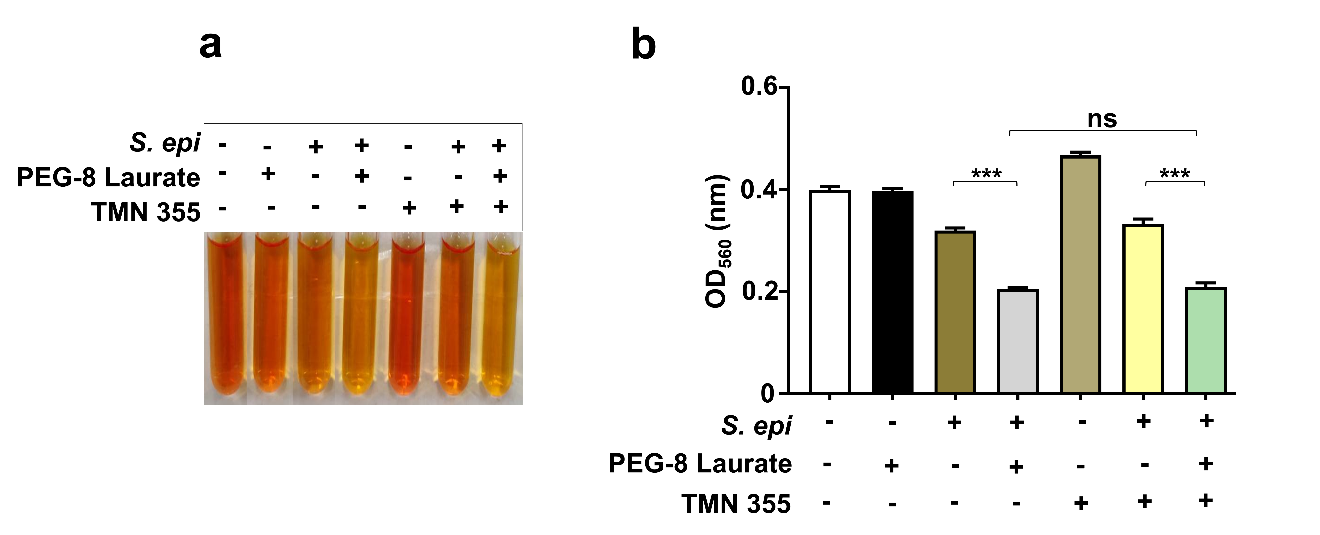


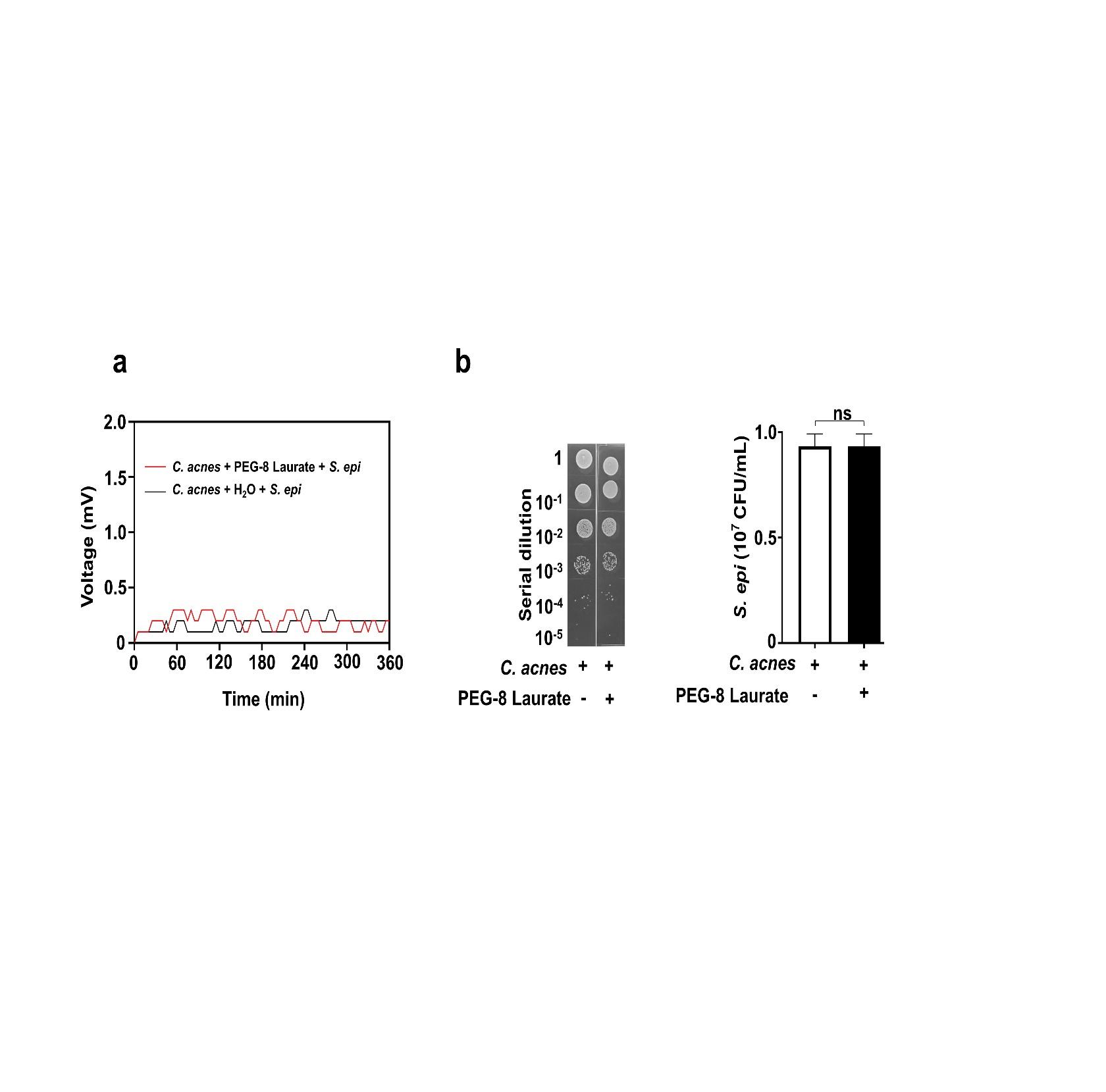
**Supplementary Fig. S3.**

**Supplementary Fig. S4.**

**
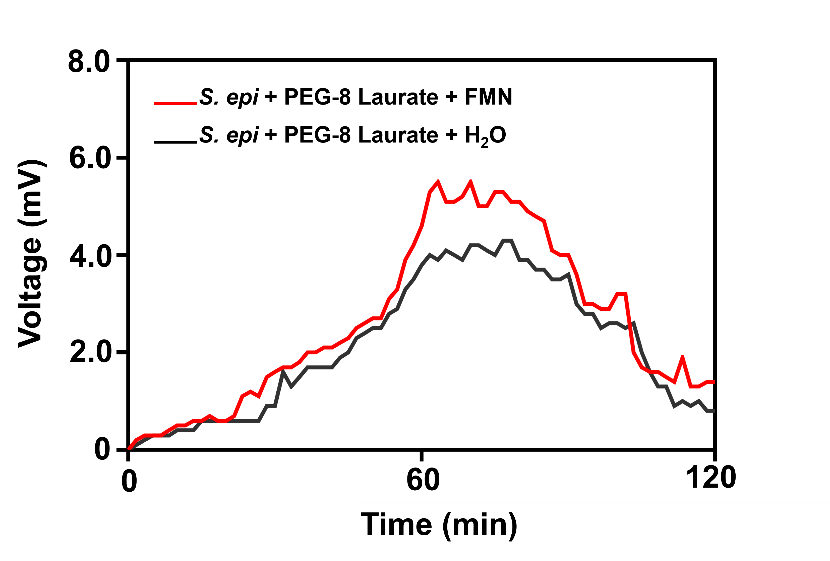
**

**Supplementary Fig. S5.**


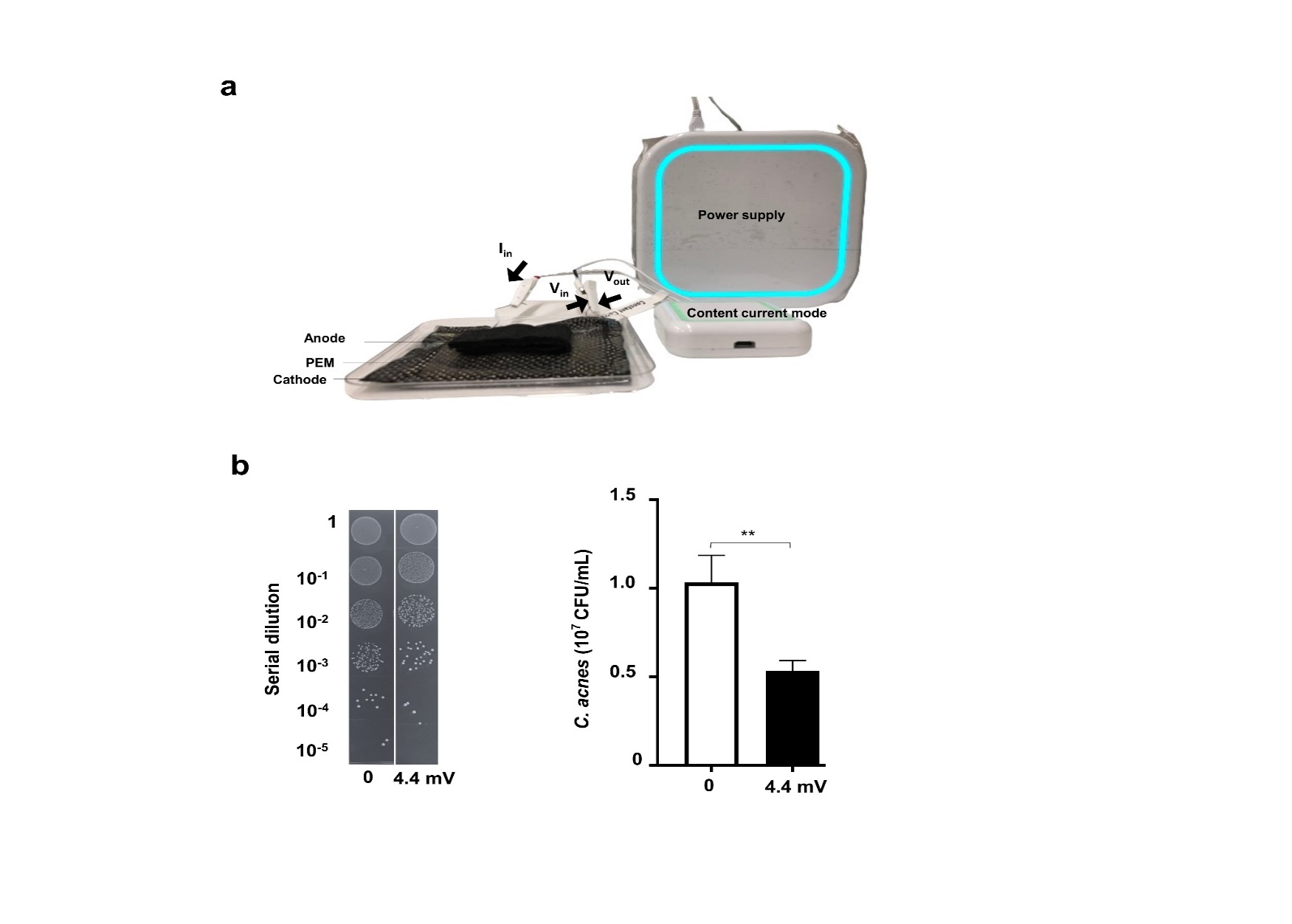

Supplement: Supplementary file 1 — Supplementary Information. [file 41598_2021_91398_MOESM1_ESM.docx]
